# ‘It needs a complete overhaul…’ district manager perspectives on the capacity of the health system to support the delivery of emergency obstetric care in an urban South African district

**DOI:** 10.1080/16549716.2019.1642644

**Published:** 2019-07-31

**Authors:** Siphiwe Bridget Pearl Thwala, Duane Blaauw, Freddy Ssengooba

**Affiliations:** a Centre for Health Policy, School of Public Health, University of the Witwatersrand, Johannesburg, South Africa; b Faculty of Health Sciences, University of Swaziland, Kwaluseni, Swaziland; c School of Public Health, Makerere University, Kampala, Uganda

**Keywords:** Implementation of emergency obstetric care, district, health system, district management, quality of care, building blocks framework

## Abstract

**Background**: A high maternal mortality ratio persists in South Africa despite developments in emergency obstetric care (EmOC), a known effective intervention against direct causes of maternal deaths. Strengthening the health systems is one of the focus areas identified by the National Committee for Confidential Enquiries into Maternal Deaths in South Africa. District managers as immediate overseers of the frontline health system are uniquely positioned to provide insight into the overall health system processes that influence the delivery of EmOC.

**Objective**: We sought to identify health system enablers and barriers to the delivery EmOC from the perspective of district managers. This would potentially unearth aspects of the health system that require strengthening to better support EmOC and improve maternal outcomes.

**Methods**: Face-to-face audio-recorded key informant interviews were conducted with 19 district managers in charge of the delivery of EmOC in one urban district. Interviews were transcribed and coded. Related codes were inductively grouped into emerging themes. Deductive thematic analysis was then applied to categorise emergent themes into the WHO health system building blocks.

**Results**: Themes included a weaknesses in the organisation of health services; a high vacancy and turnover of senior management; poor clinical accountability from EmOC providers; inadequate resources (including infrastructure, staffing, and funding); and the need to improve district health information system indicators.

**Conclusion**: The functioning of the district health system was weak, affecting the delivery of EmOC. Unless staffing is effectively addressed, the health system is unlikely to reduce maternal mortality to the desired level. Coordination of EmOC services by managers needs to be strengthened to limit fragmentation of care and improve the continuity EmOC. Furthermore, a high turnover of senior leadership affects implementation priorities and continuity in the overall strategic direction of EmOC.

## Background

A strong health system is necessary to support the delivery of health services and the implementation of interventions to improve the maternal mortality ratio [1]. Emergency obstetric care (EmOC) is an important maternal health intervention effective in reducing the direct causes of maternal deaths [2–4]. However, a high maternal mortality ratio (MMR) from direct pregnancy causes persists in the sub-Saharan Africa region despite EmOC [5]. Weak health systems common in low- and middle-income countries (LMICs) have been implicated [6,7]. Health system strengthening has therefore been declared a priority by many scholars to reduce maternal mortality [8–12]. The World Health Organization (WHO) structures the health system into six concrete building blocks. These are: leadership and governance; health services delivery; health information; medical products, vaccines, and technologies; financing; and health workforce [1]. The framework has been widely used and provides a unifying language to understand and conceptualise health systems [13,14].

The leadership and governance building block is concerned with effective oversight and stewardship, making it an important building block for health policy governing service delivery [5]. It is the overarching building block that coordinates the others [7,12]. Good governance is critical for health system performance including the reduction of maternal and child mortality [7,12,15]. For example, Rwanda, a country that met its millennium developmental goal of MMR reduction, attributes its success largely to a strong commitment to improving leadership and governance [9,16–19]. While provision of the strategic direction and vision is usually situated at the senior levels within ministries of health and departments, decentralisation of roles and power to sub-national levels where policy implementation actually occurs is important to drive health system performance [20–22]. Positioning the role of leadership and governance at the point of health service delivery means district health managers are important stewards. They provide oversight to ensure that the elements of the district health system work together to achieve common goals [1,23]. District managers are thus uniquely positioned to understand the overall processes of health system functioning at sub-national level. Management perspectives of the strengths and weaknesses of the health system in implementing maternal health policies can provide useful insights into the state of the district health system and its capacity to support the delivery of EmOC.

In South Africa, the MMR remains high at 138 per 100 000 live births, despite EmOC and other interventions aimed to reduce it [24]. The National Confidential Enquiry into Maternal Deaths (NCCEMD) has identified five priority areas requiring attention inorder to reduce the MMR in South Africa. These are haemorrhage, hypertension, HIV, health worker training, and health systems strengthening. While it is clear what needs to be done for the first four priority areas [25–27], strategies to address health systems strengthening to improve maternal outcomes are less clear. Scholars have identified inequity in resource allocation in health facilities [28]; shortage of health providers [29,30]; shortage of basic EmOC services [31]; and substandard leadership [30,32] as some of the broad health systems problems that influence poor health outcomes in South Africa. We sought to explore and describe health systems weaknesses that specifically affect the delivery of EmOC from the perspective of district managers. This will potentially unearth health systems issues that limit improvements in the MMR of the district and help identify local solutions to strengthen the health system. This study was part of a larger body of work that investigated the link between the health system and maternal health outcomes in South Africa and Rwanda (MHSAR) [9,33–36].

## Methods

### Setting

Data collection took place in one urban district of the Gauteng Province. The province has five districts. The district under study is the economic hub of the province with vibrant industrial activity that attracts migrants looking for labour, including low-skilled jobs. It is therefore densely populated, with about 3.2 million people living in approximately 1 975.31 km^2^ [37]. Informal settlements and poverty are common, challenging the public healthcare system with an increased demand for childbearing services, including EmOC. The MMR of the study district is estimated at 202 per 100 000 live births while the provincial MMR for the province is 148 per 100 000 live births [11], making it a priority district in efforts to reduce the MMR for Gauteng. About 26% of the district population access private health care mostly through private health insurance [38]. This leaves 74% of the population dependent upon public healthcare services funded by the government [38]. Public health care is administered at the district level jointly by the provincial government and the municipality (local government).

### Research design

A qualitative research design was used to explore and describe experiences and perceptions of district managers as they oversee the delivery of EmOC services in the district health system.

### Participants

A total of 19 senior managers were purposively selected from the study district. Inclusion criteria were people in leadership positions that and could make decisions that governed EmOC services. These were senior managers in the district directorate, health facilities (community health centres (CHCs) and hospitals), members of the district clinical specialist team (DCST), as well as the provincial managers that directly governed maternal health services in the district. Seven (36.8%) of the participants were male and 12 (63.2%) were female aged between 45 and 62 years. They were either medical doctors or nurse professionals.

### Procedure

Face-to-face key informant interviews were conducted in English with individual participants in the privacy of their offices at times designated by them. Interviews took about an hour on average, depending on interpersonal participant dynamics. Participants were initially asked to relate their experiences of the health system as managers, including perceived failures and successes. The following open-ended question was asked to open the discussion;

“Please tell me about your experiences as a manager that also oversees EmOC in the district. What are some of the challenges you experience?”

The health system building blocks framework was used to guide probing questions and tease out discussions. The researcher used active listening to encourage participant discussions during interviews. All interviews were conducted in English and recorded. Field notes were also taken by the researcher to document participant non-verbal cues such as body language during interviews. Findings were validated in a feed-back workshop at the district.

### Analysis

Thematic content analysis was used to first inductively identify themes and then deductively categorise them into the broader themes of the health system building block framework [1]. Thematic analysis allows flexibility enabling both inductive and deductive coding. The process starts by identifying variables as they emerge from the data [39]. It then progresses to creating broader categorisations and ultimately theory application [39,40]. Audio-tapes were transcribed and typed in MSWord. Phrases and words were coded from the transcripts that gave participant impressions, perceptions, and experiences of the health system. This first step of analysis was done inductively, without reference to researcher preconceived ideas or classifications [41]. From the transcripts, we looked for clues regarding the overall state of the district health system as perceived by managers. We also looked for barriers or bottlenecks encountered during EmOC governance and services delivery, problem-solving or troubleshooting strategies, innovations employed by managers, and identified successes or failures. We grouped related words into emerging themes. Emerged themes were then deductively categorised using the six building blocks of the WHO health systems framework [1]. To assure reliability, an experienced health systems researcher reviewed transcripts and the coded themes to confirm inter-coder agreement [41]. Field notes were also scrutinised thematically and added to themes identified from verbal transcriptions.

## Results

Table 1 shows themes identified and classified into the health systems building blocks framework.

**Table 1.** Analytical themes related to the health system building blocks.

Health system building blocks framework	Emerged themes
1. Organisation of EmOC services	Dual organisation of EmOC
	More health facilities for EmOC required
2. Leadership and governance	Clear vision guiding all managers
	Poor coordination of EmOC and fragmented care
	Duplication of management efforts
	High vacancy and turnover of senior management overseeing EmOC
3. EmOC staffing challenges	Acute shortage of skilled EmOC providers
4. Finance	Budgetary constraints failing implementation of new management plans
	Lack of funding a barrier to recruitment of senior managers
5. Drugs, equipment, and supplies	Procurement challenges frustrating managers
6. Health information	Need for improvement of health systems indicators
	Data clerks needed to administrate patient files in health facilities

### Health services delivery and organisation of health services

#### Dual organisation of EmOC

EmOC was delivered through two separate and parallel arms of government, namely the provincial government and local (municipal) government. These two arms of government were commissioned and funded by national government to deliver health care to the district population without health insurance. Local government clinics referred women to provincial CHCs and hospitals for EmOC, yet there was no opportunity for dialogue or collaboration between these two arms of government. The result was frustration of provincial managers who felt that bottlenecks in the delivery of EmOC could be solved if operations were planned and implemented collectively rather than separately (in silos) or if collaboration to troubleshoot problems was made possible.

“… There should not be a local government and a provincial government, only health services in one District. Because local government has its own systems and its own mandate, you [provincial government] come with yours and you clash in the system that’s what’s happening.” D7

“We need a serious overhaul of how maternal health services are organised in XYZ [province name]. You know when you do an engine overhaul … ” D9

#### More health facilities required for EmOC delivery

Managers claimed that the district did not have enough health facilities in which to deliver basic EmOC. The northern and far-eastern areas of the district were particularly underserved. In the northern area, one tertiary hospital served over 2.5 million people and had at least 16,000 births per year without a district or regional hospital to relieve it. In the far-eastern area, one district hospital was upgraded to a regional hospital without increasing its capacity. It served a dense population, including informal settlements of over a million people, and did not have a district hospital or CHC to relieve it. These facilities were particularly overwhelmed and overcrowding in labour wards was common.

### Leadership and management

#### Management issues: poor communication, duplication of management roles, and poor coordination by managers

All managers in the district were aware of the national goal to eliminate preventable maternal deaths. This provided uniformity of purpose as everyone strived to align their activities to achieve the goal. However, these efforts were undermined by poor coordination of health care, duplication of management roles, and a high turnover in senior management positions, at the province. Since EmOC services were delivered under different health programmes and managed by different directors in the district, strong coordination by managers was required to assure continuity of care. However, interviewees indicated that there were at least three coordinators in the district, who directed different aspects of EmOC and were in charge of the health system and the implementation processes within it. National government coordinated the DCST activities, the provincial EMS director coordinated all EMS services, while the head of department (HoD) coordinated all other district health programmes and primary health care where basic EmOC was housed. These three coordinators did not have formal platforms of communication to help them link up all the programmes under their care. Routine district management team (DMT) meetings did not include all health programme managers, limiting communication and collaboration. Thus, the three aspects of EmOC were not coordinated.

“*So we have no forum to communicate with them at any point as management in the District … we have the DMT [District Management Team], which is where most of the managers from deputy director upwards meet … But they [hospitals] don’t come … So that will not work because they [hospitals] don’t report to us [district].” D9*

#### Fragmented EmOC services

Different aspects of EmOC services were packaged in different health programmes and managed by different directors within provincial government in the district. For example, basic EmOC services were performed by CHCs and these were the responsibility of the primary health care (PHC) programme, while hospitals that offered comprehensive EmOC services were under the directorship of the province. The district clinical specialist team (DCST) in charge of clinical governance was under the directorship of national government. Obstetric ambulances were managed by a separate directorship (emergency services (EMS)) in the province. The result was a lack of understanding of some elements of EmOC in the different programmes. EMS, for example, was poorly understood by managers in health facilities and the district, affecting necessary collaboration between programmes in order to deliver EmOC efficiently. The managers or staff in these four aspects of EmOC did not engage or communicate directly in any way, and this negatively affected services delivery.

“… because it’s [EMS] poorly understood in the department of health, it’s not well accommodated …” D12

#### Confusion and duplication of management efforts

In addition, lack of clarity over management roles and inadequate communication between national and provincial management led to duplication of efforts. For example, the province developed EmOC protocols only to find that facilities were already implementing protocols developed by national government.

“… Now it goes back to what the question, what is our responsibility? … And so we [province] ended up developing a lot of protocols for them [health facilities], which was not necessary. Because protocols were already there [from national government], documents were already there, you know … That’s why the system is confusing in this country.” D9

#### High vacancy and turnover of senior management posts

Vacancy of senior management posts at the province and district was also a barrier to implementation of maternal health policy according to district managers. A provincial human resources (HR) department was responsible for hiring of senior provincial and district management including chief executive officers (CEOs) of hospitals. The province had not had a permanent head of department (HoD) responsible for all health programmes in the province for more than 5 years. Between 2013 and 2017, the province has had at least three different HoDs, and this affected EmOC delivery as strategies priorities changed with each HoD. Furthermore, only two out of seven hospitals (28.6%) in the district had appointed CEOs while the rest were acting (71.4%). In one hospital, for example, we (researchers) engaged with six different acting chief executive officers in a space of 3 months.

“… So that’s where the problem is, is that you don’t have a permanent head of department (HoD) … Everyone who comes, comes in with their vision ….” D9

### Human resources for EmOC

#### Too few EmOC providers to meet demand

The district had skilled EmOC providers (doctors, obstetricians, and midwives). However, they were insufficient in number to meet population demand across all facilities, and some health facilities worse off than others. Unfilled vacant posts were common in all facilities. Because of the high workload, some facilities had applied for posts in order to hire more doctors and midwives. Unfortunately, they were struggling with long and slow bureaucratic processes in the province since approval of new posts rested with the finance department, and not the department of health.

“… they [hospitals] are short-staffed [doctors]. So if one doctor reports sick then a gap opens … Just like last Monday, the gynae doctor had to do rounds until 8 o’ clock in the evening.” D8

Shortage of staff affected the quality of EmOC delivered. Problems included: newly-trained staff with limited experience left to deliver EmOC services without adequate supervision; women sometimes left unattended during care; delays in administering EmOC by providers (doctors and midwives) because they were busy with other patients; queueing of obstetric emergencies (e.g. for an intensive care unit (ICU) bed or caesarean section); and unsatisfactory monitoring of patients during care. Poor monitoring also drove up the demand for EmOC as small complications were likely develop into emergencies if not addressed early on. The following manager narrates;

“… In your station [e.g. labour ward], if the patient is booked for theatre, then the sister [midwife] has to leave her workstation unattended and go and catch that baby in theatre, even in high care, we do leave patients unattended if we are short staffed. There’s nothing we can do.” D11

#### Poor clinical accountability

EmOC providers (doctors and midwives) often displayed poor clinical accountability that negatively affected EmOC delivery according to managers. Unexplained late coming to work, absenteeism, and general poor quality care were common among staff. As a result, the district reportedly experienced frequent legal suits from patients with poor maternal and neonatal outcomes in a bid to enforce accountability. Managers resolved to introduce tougher measures to enforce accountability. These included issuing warning letters to doctors or making midwives defend their care in legal proceedings themselves. In the past, it had been customary for managers to represent midwives in hearings and law suits. However, managers stated that these efforts were often undermined by unions who made representations for EmOC providers in such a way that the disciplinary process was frustrated and became unduly tedious for managers. This difficulty discouraged managers from enforcing accountability to avoid the long processes involving unions.

“The union is very powerful in this hospital. It makes it very difficult to discipline doctors. We have the union represents the doctor under disciplinary action … then you have to attend endless meetings as management … you end up feeling like just letting it be. It’s too stressful.” D13

### Finance

#### Scarcity of funding for EmOC

Inadequate funding for health programmes hampered implementation of national health policies and the delivery of EmOC services in the district. For example, responding to overall EMS challenges and resource challenges (too few and very old ambulances, acute shortage of emergency staff, etc.) a turn-around strategy was developed for EMS, without a corresponding budget to operationalise it. This lead to implementation failure.

“In fact, it [turnaround strategy for EMS] came from the premier’s office, but it collapsed in the province when it got to the money issue. There was no funding allocated to the project, and the project we were told to reprioritise within the current under- funded budget and it hasn’t worked.” D12

“… approximately 50% of our vehicles technically need to be replaced for the whole province, you’re looking at 50% plus that needs to go, either for complete replacement or major refurbishment … if you also look at the norms and standard by government that states how many ambulances should be on the road, we are completely under-resourced.” D6

Budgeting processes were said to be one of the biggest obstacles to the delivery of EmOC services. District managers unanimously expressed frustration at the general budget structure, which did not disaggregate to a maternal health budget line item or specific expenditure at the district level. As a result, managers felt disempowered to plan and execute various aspects of the maternal health programme. They felt unable to manoeuvre budget line items. Neither did they have the room to suggest changes nor cuts without a specific maternal health budget line which could have been the basis for argument. Budgetary constraints also interfered with recruitment of staff for both replacements of retired or resigned staff and staff for newly created posts.

### Insufficient resources

Inadequate resources, including infrastructure and staff, were also a barrier to full implementation of national policies such as maternal health guidelines. For example, magnesium sulphate to manage patients with severe hypertension in pregnancy was sometimes not used because of inadequate nursing staff to monitor patients on the drug in facilities as it was considered labour intensive. Moreover, CHCs did not offer some of the prescribed basic EmOC services (e.g. manual removal of placenta) because of a lack of reliable transport to refer women safely if complications developed.

“We do not routinely use magnesium sulphate for severe pre-eclampsia as the national guidelines advise because we have an acute shortage of sisters [nurse-midwives]. It would require frequent monitoring for magnesium toxicity, but we can’t do that because of staffing ….” D17

### Insufficient funding a barrier to management recruitment

According to the government’s occupation-specific dispensation (OSD) policy, some senior management positions (e.g. chief executive officers of hospitals) were to be staffed by highly trained professionals, such as doctors. However, budgetary constraints made recruitment difficult, with district directors appointing managers in acting positions because it was much cheaper than proper appointment. As a result, most (71.6%) hospitals had acting senior managers rather than properly appointed managers. It was also difficult to appoint managers who were medically trained in the district according to policy and budgetary constraints, limiting implementation of the policy.

“… Because if you act in a post, it means … you come in and work but don’t necessarily paid by for the post as a manage … which saves money for the district.” D7

### Drugs, equipment, and supplies

Respondents indicated that health facilities were mostly able to access essential equipment, blood, and other supplies for EmOC. This is a positive aspect of the delivery of EmOC services. Whole blood and blood products were often readily available in all hospitals as expected. However, frustration with procurement procedures was common and managers felt that accountability of the procurement department was unsatisfactory and at times led to unexplained stock-outs of lifesaving drugs and supplies. Equipment such as the balloon tamponade and syntometrine (uterotonic drug) used for managing obstetric haemorrhage were an example of frequent stock-outs due or delays by the procurement department.

“The problem is the procurement process in the hospital … They send me from pillar to post … And I was thinking as a doctor all I need to say, ‘I need this equipment’, and somebody’s job is to go and source it. But I have to go and get a quotation, when you get the quotation, you wait for finance ….” D7

### Health information

The district health information system (DHIS) was viewed as a valuable tool for gathering useful information to inform planning of EmOC services. District managers suggested that the quality of data collected could be improved by hiring data clerks in all health facilities, rather than relying on non-governmental organisation (NGO)-supported clerks. These clerks had very high attrition and turnover rates as the NGO frequently ran out of funding. They gave clerks very short contracts of less than a year. This could partly curb the problems such as of lost patient files, as trained clerks are proficient in schematically filing, archiving, and retrieving information. Also, indicators collected by the DHIs could also be improved. Emergency services (EMS), for example, wanted indicators that could directly monitor the responsiveness of EMS. The existing DHIS indicator showed significant increases on the number of ambulance calls for EmOC patients to be transported from CHCs to hospitals but it was not clear if this development was positive or negative, or what the implication for the health system overall was;

“… if inter-facility demand for ambulances increases, does it mean EMS is doing well or not? … What does it mean for you as a hospital? Or what does it mean for me as EMS? If last month I moved a thousand patients from your facility to another facility and next month I move two thousand, is the service improving or deteriorating? … And for me it’s to say if I start moving every second patient, then maybe you are not providing appropriate level of care. But they [health facilities] don’t want to accept that ….” D12

## Discussion

Health services delivered in dual parallel arms of government (provincial and municipal) that did not communicate or collaborate effectively undermined the delivery of quality EmOC services. Bottle-necks in the delivery processes of EmOC across these parallel arms of government were difficult to resolve because of the lack of communication platforms and links. This affected the quality of care offered. Within provincial government, different aspects of EmOC services were packaged between different poorly coordinated health programmes resulting in the fragmentation of care. In addition, a high turnover and vacancy of senior management brought instability in the health system leadership. This disrupted management implementation plans as priorities changed with each new manager. There was also a lack of clarity of management roles between national and provincial managers, leading to duplication of efforts. Furthermore, inadequate resources were a barrier to implementation of maternal health (EmOC) policies and compromised the quality of care for EmOC.

High vacancy and turnover of senior management posts in the district are worrisome as it introduces instability in the strategic direction and implementation priorities of the health system as pointed out by managers. Duffield and colleagues [42] identify numerous health system problems associated with a high turnover of senior management. These include instability of organisations from loss of human capital, unwarranted high economic costs, compromised quality of care, and general reduction in staff morale [42]. This demonstrates the importance of leadership in driving EmOC implementation policies and strategies in the district.

Poor quality of care in health systems is associated with high maternal mortality [43,44]. Health services therefore need to be organised in such a way that delivery of care is continuous and not fragmented to uphold the quality of health systems. Strong coordination of health services by managers and integration of health services is mandatory to assure continuity of care [45]. In this study, the organisation of health services was considered complex and poorly coordinated, and this compromised the quality of the health system and corresponding quality of EmOC delivered to women. To improve integration, managers proposed positioning EMS governance within the district rather than at the national level. It then becomes fully integrated with other district health programmes for planning and reporting. This change could potentially give EMS space to discuss operational challenges, make recommendations, enable other managers to understand EMS, and streamline operations for more efficient and effective service delivery.

Donabedian proposed that quality of care is attributable to the structure (e.g. infrastructure, resources where EmOC is given); the process (e.g. assessment and treatment modalities for EmOC); and the outcomes (e.g. maternal mortality) [46]. It is therefore important to safeguard the quality of care by improving the process (e.g. coordination and integration services to assure continuity of care) in the delivery of EmOC.

Inadequate resources limit implementation of health policies aimed at improving maternal outcomes [47]. In this study, for example, the policy-prescribed drug of choice to manage severe hypertension in pregnancy was not always used when indicated because of staff shortage. Hypertension in pregnancy is one of the leading causes of maternal mortality [8]. We also observed that CHCs in the district struggled to safely give all prescribed basic EmOC life-saving procedures prescribed in the maternal health guidelines [27] as previously reported [36]. This decision although reasonable in the short term promoted centralisation of EmOC to higher levels of care [36]. Thus, primary health care (PHC) implementation in which basic EmOC services are located is also undermined. PHC is one of the key strategies endorsed to turn around the persistent MMR [48,49]. Other LMIC studies have also shown fewer basic EmOC facilities than the recommended benchmarks [31,50,51]. To ease the EmOC burden on EMS and hospitals in the short term, all CHCs need to be provided with requisite resources to be fully functional basic EmOC facilities [27,52]. This could potentially relieve hospitals by decentralising the currently centralised basic EmOC services [36]. In the long term, policymakers may need to consider adding health facilities, to relieve hospitals overwhelmed by a high demand for EmOC [52,53].

Finally, acute shortages of EmOC providers (doctors and midwives) persist and continue to undermine the district health system’s ability to provide EmOC timeously and on demand [36]. To put it into perspective, a recent study on EmOC preparedness in the same district showed that only 12.5% CHCs and 42.9% hospitals were able to provide skilled providers for EmOC 24 h a day, 7 days a week [36]. Similarly, scholars identify a shortage of health providers as one of the major health system weaknesses in LMICs [31,54,55]. The South African government has introduced policies such as the occupation-specific dispensation policy (OSD) aimed at improving retention of skilled health providers in districts through financial incentives [56,57]. Unfortunately, health system challenges like insufficient funding for remuneration of highly skilled health providers and senior managers, limits the implementation of OSD making it difficult to resolve the staffing problem. More needs to be done to aggressively increase staffing numbers to meet the demand for EmOC as part of health strengthening initiatives.

## Conclusion

Overall, managers perceived the district health system as weak and this undermined the quality of EmOC delivered to women. To improve the quality of care delivered by the district health system, the way in which health services are currently organised will have to be reviewed. Coordination of health programmes needs to be improved to enable continuity of EmOC. Lack of stability in the district leadership responsible for the strategic direction as well as directing implementation of EmOC policies is a critical gap that needs a prompt address. Furthermore, the persistence of acute staff shortage considerably compromises the quality of EmOC delivered.

## Paper context

Health systems strengthening is one of the five high-priority areas requiring focus to reduce the MMR in South Africa [8]. However, it is unclear what about the health systems needs strengthening or how. This study investigates health system weaknesses that have a bearing on the delivery of EmOC from the perspectives of district managers as immediate overseers of implementation at sub-national level. Findings thereof will inform a tailor-made health system strengthening initiative for the district.
